# Cryptic species of *Aspergillus* section *Terrei* display essential physiological features to cause infection and are similar in their virulence potential in *Galleria mellonella*

**DOI:** 10.1080/21505594.2019.1614382

**Published:** 2019-06-06

**Authors:** Michaela Lackner, Judith Obermair, Verena Naschberger, Lisa-Maria Raschbichler, Carmen Kandelbauer, Johannes Pallua, Julia Metzlaff, Sibylle Furxer, Cornelia Lass-Flörl, Ulrike Binder

**Affiliations:** a Division of Hygiene and Medical Microbiology, Medical University Innsbruck, Austria; b Department of Pathology, Medical University Innsbruck, Austria

**Keywords:** Invertebrate *in vivo* model, *Galleria mellonella*, *A. terreus* species complex, hypoxia, temperature adaptation, accessory conidia

## Abstract

*Aspergillus* species account for the majority of invasive mold infections in immunocompromised patients. Most commonly, members of the *Aspergillus* section *Fumigati* are isolated from clinical material, followed by isolates belonging to section *Terrei*. The section *Terrei* contains 16 accepted species. Six species were found to be of clinical relevance and studied for differences in growth adaptability and virulence potential. Therefore, a set of 73 isolates (22 *A. terreus* s.s., 8 *A. alabamensis*, 27 *A. citrinoterreus*, 2 *A. floccosus*, 13 *A. hortai*, and 1 *A. neoafricanus*) was studied to determine differences in (a) germination kinetics, (b) temperature tolerance, (c) oxygen stress tolerance (1% O_2_), and (d) a combination of the latter two. Virulence potential of phialidic (PC) and accessory conidia (AC) was studied in *G. mellonella* larvae, using survival as read out. Further, the formation of AC was evaluated in larval tissue. All isolates were able to grow at elevated temperature and hypoxia, with highest growth and germination rates at 37°C. *A. terreus s.s., A. citrinoterreus*, and *A. hortai* exhibited highest growth rates. Virulence potential in larvae was inoculum and temperature dependent. All species except *A. floccosus* formed AC and germination kinetics of AC was variable. Significantly higher virulence potential of AC was found for one *A. hortai* isolate. AC could be detected in larval tissue 96 h post infection. Based on these findings, cryptic species of section *Terrei* are well adapted to the host environment and have similar potential to cause infections.

## Introduction

Members of the genus *Aspergillus* are among the most common fungal opportunists found in human and animal infection [1,2]. The monophyletic genus *Aspergillus* currently contains 339 species [3]. The minority of these species were reported from human infections and are associated to the sections *Fumigati* [4], *Flavi* [5], *Nidulantes* [6], *Usti* [7], *Cremei* [8], *Nigri* [9] and *Terrei* [9,10]. Based on molecular phylogenetic studies, the previous morphological species *A. terreus* was split into 16 accepted species within the newly introduced section *Terrei: A. terreus sensu stricto* (s.s.),*A. alabamensis, A. allahabadii, A. ambi-guus, A. aureoterreus, A. carneus, A. floccosus, A. hortai, A. microcysticus, A. neoafricanus, A. neoindicus, A. niveus, A. citrinoterreus, A. bicephalus, A. iranicus, and A. pseudoterreus* [9]. Even though the species within the section *Terrei* are taxonomically accepted species, they are referred to as cryptic species. Each cryptic species forms a distinct phylogenetic clade, but these are indistinguishable by morphological features. Cryptic species can only be reliably identified using molecular identification methods. In addition species boundaries based on the capability of producing fertile offspring that would be needed to define a biological species are often missing for anamorphic fungi [11,12]. Numerous cryptic species and species complexes haven been recognized since the molecular era of phylogeny, causing a lot of dynamics in the field [13–15]. Although *A. terreus sensu lato* (s.l.) is geographically widespread, clusters with particularly high *A. terreus* infection incidences were reported from Innsbruck (Austria) and Houston (USA) [16]. Approximately 5.2% of all aspergillus infections are caused by *Aspergillus terreus* s.l [17].

Disease entities, risk factors, and affected patient cohorts do not differ between *A. fumigatus* and *A. terreus*, but treatment differs between the two etiological agents, while amphotericin B is active against *A. fumigatus* it fails to exhibit activity against *A. terreus* [18,19]. Therefore, primary treatment for the majority of patient cohorts is based on triazoles [20]. But, similar to *A. fumigatus*, posaconazole resistance is emerging in *A. terreus* with resistance rates ranging from 0% to 13.7%, depending on geographical origin and patient cohort [21].

A morphological feature shared only by few human pathogenic aspergilli, is the formation of accessory conidia (AC), also called aleuroconidia. The majority of aspergilli producing AC are members of the section *Terrei* [18]. AC were shown to be distinct from phialidic conidia (PC). Both types are produced asexually. The production of PC includes the formation of conidiophores, on which conidia arise from phialides. In contrast, ACs are produced by accessory cells emerging from vegetative hyphae, as an alternative way of dispersal and outlast in challenging conditions. AC were produced *in vitro* and within the host, but the fungi usually fail to produce PC during infection [22]. Differences were detected in their outer cell surface, e.g. exposing beta-1,3-glucan patches, in their metabolic activity, germination kinetics and ergosterol content [22]. Further, AC were shown to cause increased inflammatory response in mouse macrophages and in infected mouse lungs compared to PC [23]. The formation of AC *in vivo* was postulated to be one possible reason for the high proportion of disseminated infections and the fulminant disease progression of *A. terreus* infection compared to other *Aspergilli* [24,25]. However, their impact on dissemination and virulence potential remains to be fully understood. Another hypothesis that might facilitate dissemination of *A. terreus* is underlined by the ability of *A. terreus* conidia to survive within immune cells (macrophages and dendritic cells) of healthy individuals. Possibly, these cells serve as a vehicle for conidia to reach other body regions [26,27].

The formation of AC *in vivo*, let us assume to influence virulence potential of *A. terreus* species in the wax moth, visible in the progression and outcome of infection. Further, the differences in their surface could also lead to different immune response and therefore differences in killing ability of larvae. Therefore, we investigated differences in virulence potential between the different cryptic species and the two types of asexually formed conidia (AC and PC). Moreover, we studied differences between the cryptic species and their adaptability to (a) high temperature, (b) nutrients, (c) oxygen stress (1% O_2_), and (d) a combination of the latter two.

## Results

### Aspergillus *section* Terrei *isolates are well adapted to 37°C, nutrient conditions in the host and oxygen stress*

**Figure 1.**
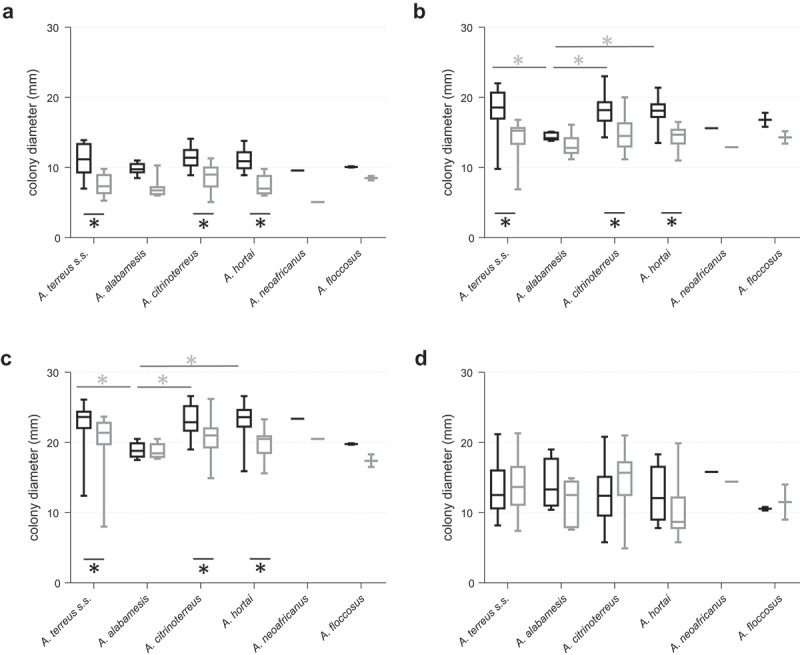
Radial growth of cryptic species within section *Terrei* differs depending on medium and incubation temperature. Box and whisker blots represent the median ± maximum/minimum colony diameter of strains grown on ACM (black boxes) and RPMI_1640_ (grey boxes), respectively, at 25°C (a), 30°C (b), 37°C (c) and 42°C (d) for 48 h. Stars below boxes indicate significant difference between ACM and RPMI_1640_ medium (*p* < 0.05; 2 Way ANOVA, Sidak´s multiple comparison). Grey stars above boxes indicate significant difference in the median colony diameter between the species (*p* < 0.05; 2 Way ANOVA, Tukey´s multiple comparison).

We studied species-specific differences in essential host related physiological features, including growth and germination rates at 37°C (essential physiological switch from resting to active stage), temperature tolerance (range 25–42°C), adaptability to low nutrient conditions (rich ACM medium versus RPMI_1640_) and oxygen stress (1% O_2,_ mimicking oxygen depletion in infected/necrotic tissue) [28]. Growth rates of all tested cryptic *Terrei* species were temperature and medium dependent; highest average colony diameters were found at 37°C on rich medium (ACM) (Figure 1). Significant (*p* < 0.05) smaller colony diameters were evident at 25°C and 42°C for all species (Figure 1). Also at 30°C all species exhibited lower growth, although not statistically significant. The three clinically most common species [17,29] *A. terreus s.s., A. citrinoterreus* and *A. hortai* showed superior growth rates (statistically significant, *p* < 0.05) at temperatures relevant for human infection, 30°C (superficial infections) and 37°C (deep infections), when compared with other, less abundant species of the section *Terrei*. Compared to growth at 37°C and 42°C, standard deviation of colony diameters of cultures grown at 25°C is very low between the individual strains. This might indicate that the adaptation to temperatures found in mammalian hosts is strain-specific, while growth rates at ambient temperatures up to 25°C are very preserved and consistent. The highest temperature tested, 42°C, seem to be the most challenging temperature for all species, resulting in high variations of colony diameters in parallel experiments of the same strain and between different strains of the same species.

Although growth was still reasonably well on nutrient limited media, RPMI_1640_, *A. terreus s.s., A. citrinoterreus* and *A. hortai* grew significantly (*p* < 0.0001) better on ACM medium, than on RPMI_1640_ at all temperatures except 42°C, where variability was high.

Not only low nutrient availability might limit growth in the human host, but also low oxygen concentrations. Therefore, growth of all *Terrei* isolates was compared between hypoxia (1 % O_2_) and ambient oxygen concentration (21%) at human body temperature (37°C). All cryptic species were able to grow when exposed to the combination of hypoxia and 37°C. Germination at these conditions is an essential feature to cause infections in the human host, since, the resting conidia change to an active, potentially invasive, stage. Average colony diameters were significantly (*p* < 0.05, Multiple *t* test) lower in hypoxic conditions when compared to normoxic conditions, which corresponds to our previous observations (Table 1) [30]. At 48 h, growth reductions due to hypoxic conditions was most pronounced for *A. floccosus* and *A. hortai* (Table 1). At later time points, differences became less distinct (except for *A. neoafricanus* (*n* = 1), indicating that the reduced growth was mainly linked with delayed germination. Highest range of colony diameters in hypoxia was detected for *A. citrinoterreus* isolates (Table 1), evident by highest standard deviation.

**Table 1.** Radial growth of *Terrei* under hypoxic and normoxic conditions. Fungi were grown on ACM at 37°C and colony diameters were determined at 48 h and 72 h. Average was calculated from colony diameters of three biological replicates of all strains of the respective species. Growth reduction presents relative growth reduction of cultures grown in hypoxia. For *A. neoafricanus* average was calculated from repeated results of the same strain, indicated by *.

	48 h							72 h						
		Colony diameter [mm]							Colony diameter [mm]					
*Species*	Growth reduction [%] in hypoxia	Range	Normoxia Average	SD	Range	hypoxia Average	SD	Growth reduction [%] in hypoxia	Range	Normoxia Average	SD	Range	Hypoxia Average	SD
*A. terreus s.s.*	23.3	12.4–26.1	22.8	3	10.2–19.9	17.5	2	17.7	16.7–45.7	37.6	5.7	23.8–34.1	31	2.5
*A. alabamensis*	18.6	17.5–20.5	18.9	1	13–17.5	15.4	1.6	13.5	27.4–35.3	30.6	2.6	22.9–29.9	26.5	2.2
*A. citrinoterreus*	24.3	19–26.6	23	2.2	7.3–21.7	17.5	2.9	20.4	29–45.3	38.6	4.6	10.2–36.8	30.7	5.2
*A. hortai*	23.3	15.9–26.6	22.9	3.1	13–20.8	17.1	2.3	21.2	26.3–46.2	37.7	5.4	24.5–35.8	29.7	3.9
*A. floccosus*	23.3	19.7–19.9	19.8	0.2	14.3–15.3	14.8	0.2	23.0	28.2–31	29.6	0.2	22.5–23.1	22.8	0.2
*A. neoafricanus*	20.9		23.4	1.2*		18.5	0.3*	22.9		40.7	2.1*		31.4	1.6*

### Germination kinetics were strain-specific rather than species-specific and is particularly stimulated by 37°C

Germination rates of phialidic conidia (= spores) were determined for each isolate after 8 h of incubation at 30°C (body surface temperature) and 37°C (body core temperature) (Table 2). At that time point, the percentage of spores showing germ tube formation were higher at 37°C, which correlates to what has been previously reported [31]. Highest rate of spores with germ tubes was detected for *A. citrinoterreus* (56.6% ± SD 2.2). The majority of *A. citrinoterreus* strains exhibited high germination efficiencies (>50% of all conidia changed to the active stage), followed by *A. alabamensis* and *A. terreus s.s*. (48% ± SD 1, 24.4% ± SD 2.4) at 37°C. Similar germination efficiencies were found for 30°C. Overall, great variety was observed in germination rates ranging from 3%–100% at 37°C. Interestingly, *A. floccosus* strain number 204 (clinical isolate) had an germination efficiency of 100% at both 30°C and 37°C with germ tubes by far extending the spore diameter, while the strain obtained from the culture collection had a germination efficiency as low as 13% after 8 h incubation (Table 2). Interestingly, the higher germination efficiency did not result in higher colony diameters in the two dimensional growth assays, nor in increased virulence potential. A limitation for this species was that we only had two isolates available – one clinical isolate of *A. floccosus* and the neotype strain from KNAW culture collection – so the data need to be interpreted with caution. Possible explanation for the observed differences in germination efficiency might account to the origin of the neotype strain, which is an environmental isolate, or the physiological changes (degeneration, aging) during long-term storage of the neotype strain [32].

**Table 2.** Germination rates of *Terrei*. 10^5^/ml phialidic conidia of each isolate were incubated in ACM and number of germinated cells evaluated microscopically. Germination rates express the percentage of conidia with germtube formation normalized to the total number of counted conidia (*n* = 100) after 8 h of incubation at 37°C and 30°C. Average germination rates and mean germination rate were calculated from data of three independent experiments of all strains tested per species.

Species	A. terreus s.s.		A. citrinoterreus		A. alabamensis		A. hortai		A. neoafricanus		A. floccosus	
	37°C	30°C	37°C	30°C	37°C	30°C	37°C	30°C	37°C	30°C	37°C	30°C
Average germination rate [%]	28.4	6.05	56.6	19.5	48.0	19.2	25.4	4.2	9.3	0	56.5	54
Range [%]	10–76.3	0–19.7	3–97	0–87.3	17–91.3	2–79.7	5.3–69	0–14.3			13–100	8–100
[%] strains with germination rate above 50%	14.3	0	55.6	18.5	37.5	12.5	7.7	0			50	50
Median germination rate	17.5	4.17	63.7	8.7	38.5	11	17.0	3.7				

### *All cryptic species except* A. floccosus *form accessory conidia which differ from phialidic conidia in germination properties in a strain dependent manner*

**Figure 2.**
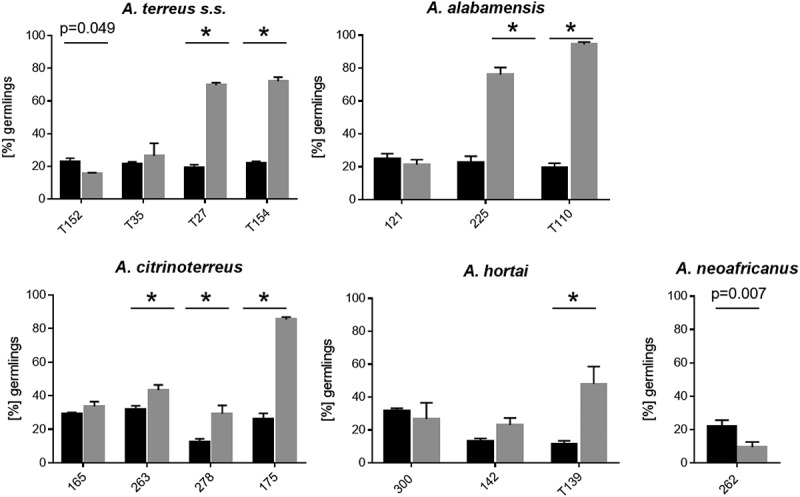
Germination rates of accessory conidia (black bars) was compared to phialidic conidia (grey bars) for selected strains incubated at 37°C for 8 h. Bars represent average germination rate of three independent experiments. Error bars represent standard deviation. Significant differences in germination rates of accessory conidia from phialidic conidia are indicated by * (*p* < 0.0001, Sidak´s multiple comparison, unless otherwise indicated).

In a comprehensive study by Deak et al. [22] it was shown that all 100 *A. terreus* isolates tested formed AC, therefore, only a small strain set (*n* = 15) was used here to check for inter-species varieties. Except for the two *A. floccosus* strains, all strains tested were able to form AC which could be harvested for further investigations. This corresponds to the species description of *A. floccosus*, which does not mention the ability to form AC either [9]. Germination rates were strain dependent for both types of conidia. For the majority (55.3%) of strains tested, germination rates of AC were significantly lower than those of PC. In 33.3% of the tested strains no differences in germination rates between AC and PC were observed, and only 13.3% of all strains exhibited significantly higher germination rates for AC than PC (Figure 2).

### *All cryptic species exhibit similar virulence potential in* G. mellonella, *detected differences were strain dependent rather than species dependent*

**Figure 3.**
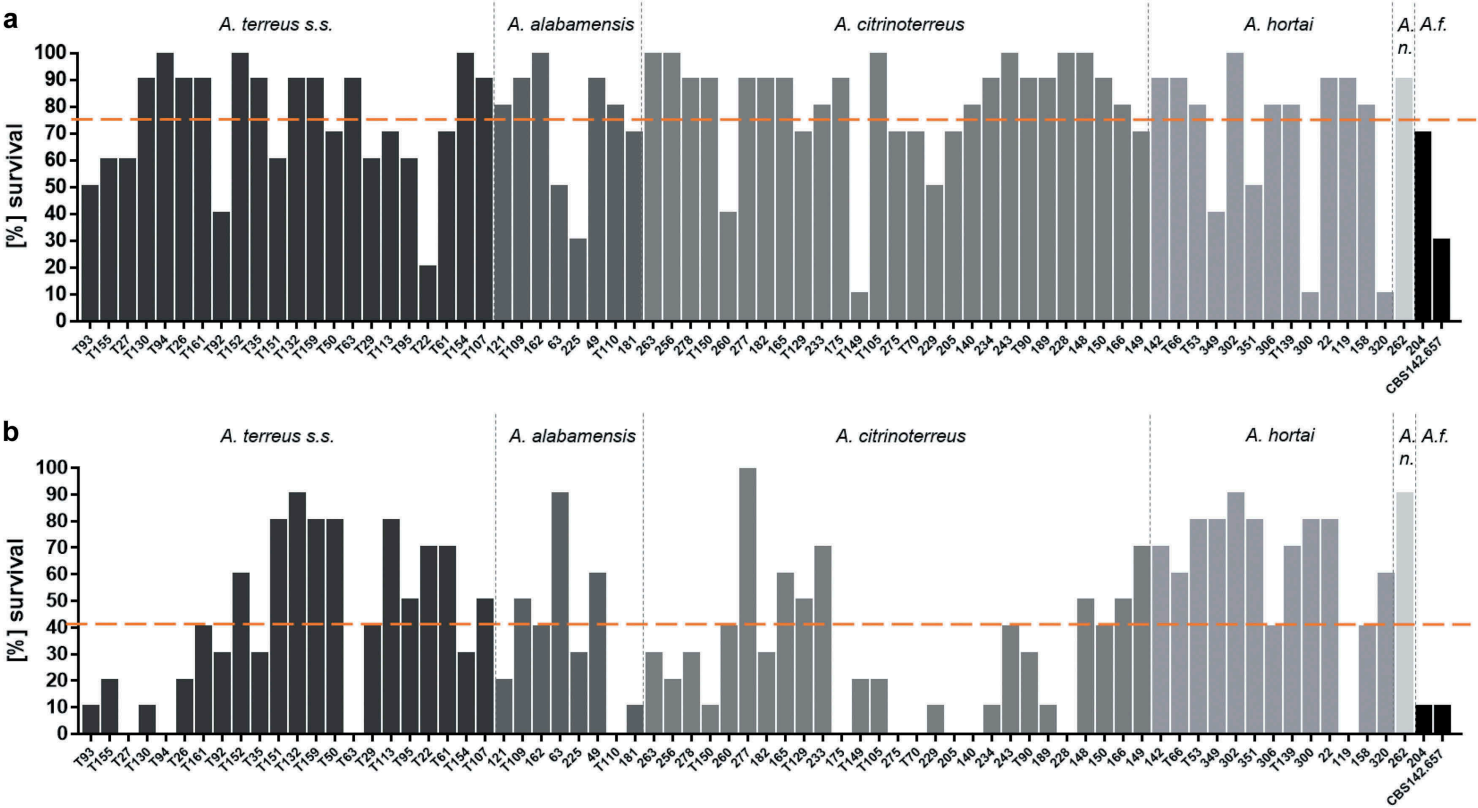
Survival rate of *G. mellonella* larvae 48 h after infection with 10^7^ phialidic conidia of each isolate, respectively, and incubation at 30°C (a) and 37°C (b). Bars represent average survival rate of three independent experiments (60 larvae in total). Dotted horizontal line represents average survival rate of all 73 strains tested at 48 h (75% at 30°C and 41% at 37°C).

**Figure 4.**
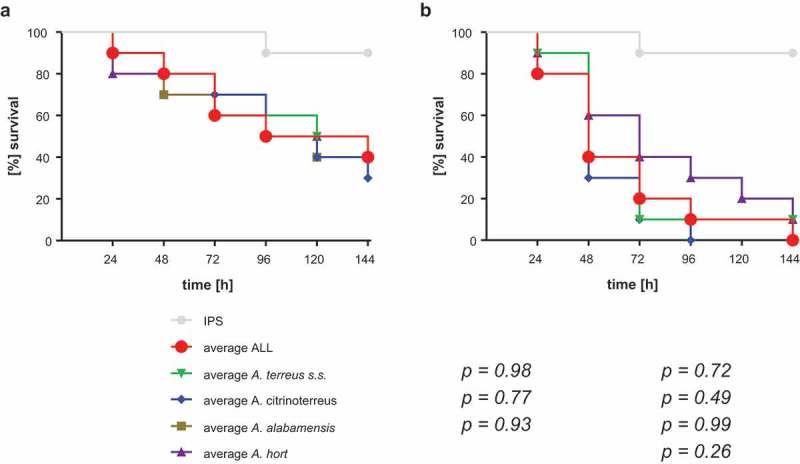
Average survival rates per species at 30°C (a) and 37°C (b). Average survival rates were calculated from all isolates per species, all three independent experiments. Average ALL represents the average survival rate of all 73 tested isolates. Kaplan-Meier curves were analyzed for significance by Log-Rank (Mantel Cox) test, resulting in no significant difference of any of the curves to the average survival rate of all 73 strains (*p* values are indicated).

All 73 strains were tested for their virulence potential in the alternative host *G. mellonella* to decipher inter- and intraspecies variations. Larvae were injected with either 10^6^ or 10^7^ conidia, based on our previous study, where these concentrations where shown to be ideal for testing *A. terreus* isolates, and incubated either at 30°C or 37°C to check for temperature dependent killing. All isolates tested were able to cause disease and subsequently death in *Galleria* larvae. From previous experience, 48 h were chosen as an ideal time point to compare for difference in virulence potential. At both temperatures virulence potential in *Galleria* larvae was highly variable and strain dependent rather than species dependent (Figure 3). Similar to a previous study testing only few isolates [31], mortality rates in larvae incubated at 37°C was higher than at 30°C, correlating to higher germination rates of all species at 37°C compared to 30°C. At 37°C and 48 h post infection, the majority of *A. citrinoterreus* isolates revealed mortality rates above the average mortality rate of all 73 strains. In contrast, the *A. hortai* group caused survival rates that were below the average. However, averaged 6-day survival curves of each species, showed similar survival when compared to the collective survival average of all 73 strains (Figure 4). Further, none of the average survival curves was significantly different from each other at any temperature (Log-Rank test). A more detailed analysis, comparing individual Kaplan Meier curves to the average survival rate revealed that only 4 of all 73 isolates caused significantly higher than average mortality to the larvae at 37°C (Fig. S1). These were *A. alabamensis* T110 (*p* = 0.02), *A. hortai* 119 (*p* = 0.01), and *A. citrinoterreus* T150 and 175 (*p* = 0.01; *p* = 0.002). Three isolates, all belonging to *A. hortai*, resulted in lower virulence potential than the average (T53, *p* = 0.04; 349, *p* = 0.01; 302, *p* = 0.01). This finding correlated to the lower mortality rates of *A. hortai* seen at 48 h post infection (Figure 3(b)) and the higher, although not significantly different, average survival rate (Figure 4).

Overall, no correlation (Pearson correlation) between germination efficiency or growth rate of the individual isolates and the virulence potential (median survival) was found. Neither, could strains with higher virulence potential than the average be correlated to the source of isolation (body site or country), which is understandable regarding the small number of strains that showed significant difference.

### Virulence potential of accessory conidia is variable

**Figure 5.**
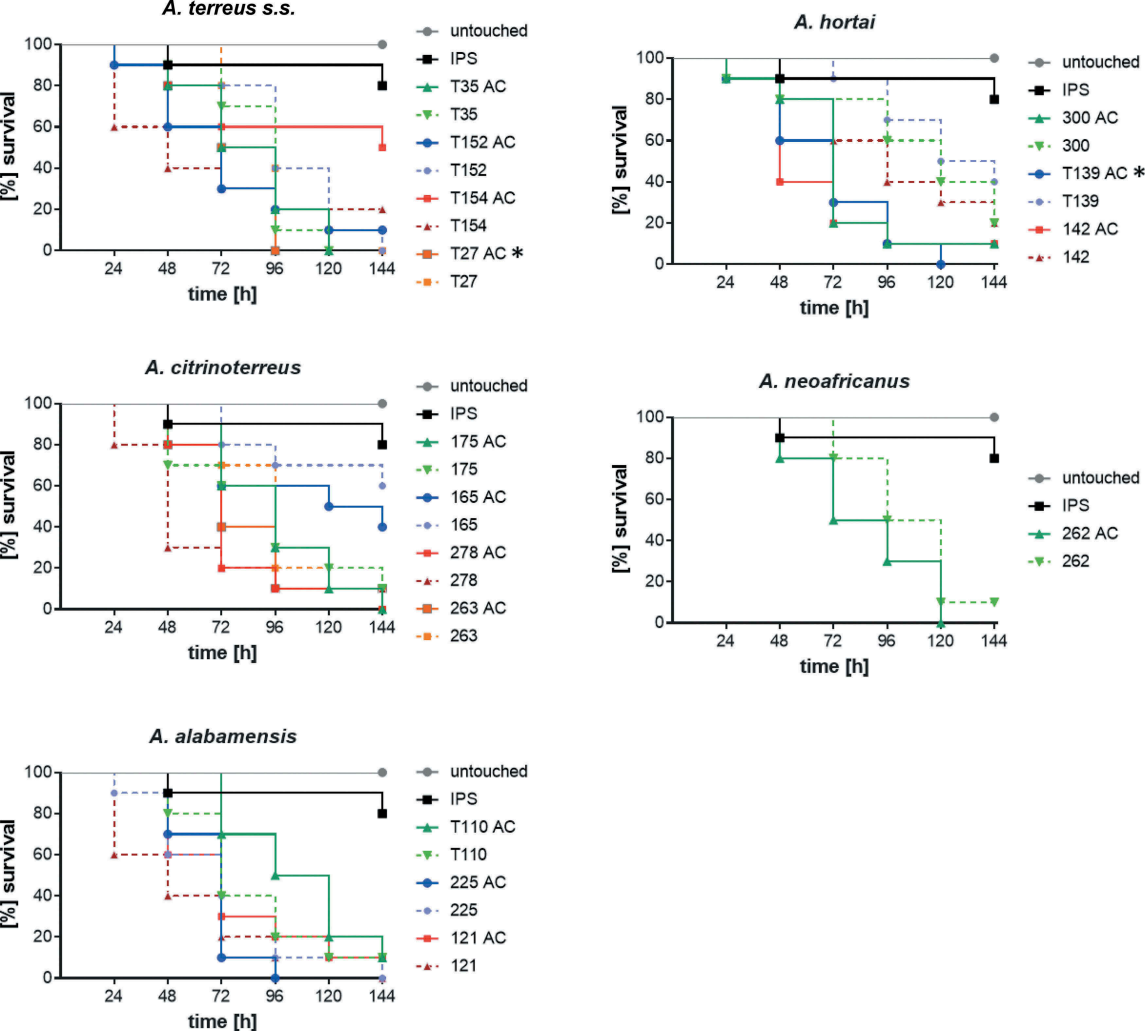
Virulence potential of accessory conidia versus phialidic conidia of selected strains. Larvae were infected with 10^6^ conidia of the respective strain and incubated at 37°C for six days. Kaplan-Meyer curves represent average survival of three independent experiments (60 larvae in total). Accessory conidia of strain T154 and T24 caused significant lower survival rates than phialidic conidia (according to Log-Rank test (*p* < 0.05); indicated by*).

It has been postulated that inflammatory response to AC is superior to PC and that the formation of AC *in vivo* could contribute to higher likelihood of dissemination within the body. Additionally, because of their superior germination kinetics shown in a previous study, they might be more virulent than PC [22,23]. To confirm this in the cryptic *A. terreus* species, virulence potential of AC and PC was compared in *G. mellonella*. The same strain set as for evaluating germination kinetics was chosen. To make sure, differences were not overlain by too fast killing of larvae, the inoculum size was reduced to 10^6^ conidia/larva and larvae were incubated at 37°C. Overall, survival rates obtained for larvae infected with AC were lower than of PC, but significance was only reached for one strain (*A. hortai* T139, *p* = 0.001; Log-Rank) (Figure 5). Negative correlation was found to germination kinetics in this case, as in this strain significantly less AC exhibited germ tubes at 8 h than PC.

**Figure 6.**
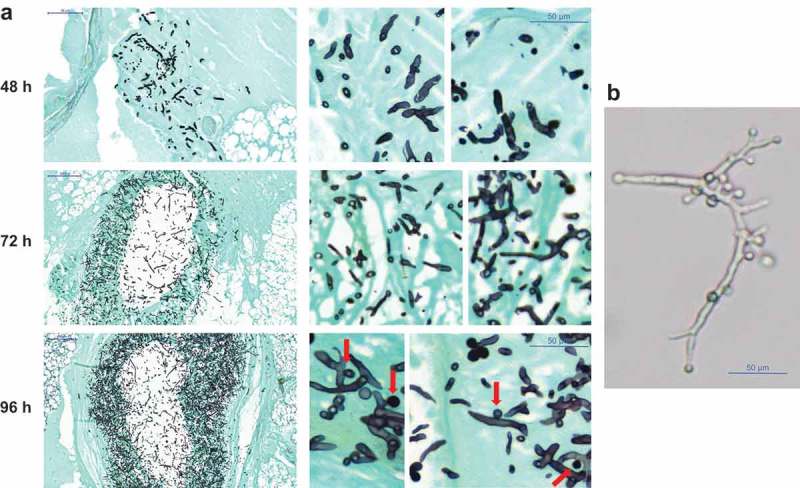
(a) Histopathology of *G. mellonella* larvae infected with 10^6^ phialidic conidia of *A. hortai* 142. Larvae were infected, incubated at 37°C and sacrificed at the indicated time points. To simplify recognition of fungal elements samples were stained with Grocott´s silver stain. (b) Presence of AC on *A. hortai* 142 hyphae grown at 37°C in liquid Sabouraud medium for 5 days.

AC formation was previously detected in murine models [33], therefore, histological preparations of a small set of larvae were evaluated for AC formation which were detected in only few samples, for example *A. hortai*. Our results suggest that also in *G. mellonella* AC are formed at a later stage of infection, as in none of the specimen investigated at 72 h or earlier AC were detected, but were found in 96 h samples (Figure 6).

## Discussion

Invasive infections in humans are limited to a small set of species within the genus *Aspergillus. Aspergillus fumigatus* causes the fast majority of infections among all aspergilli [19,34]. Although *A. terreus s.l*. infections are less frequently found (approx. 5% of all *Aspergillus* infections), they are: (a) associated with higher rates of dissemination, (b) therapeutically more difficult to address due to their intrinsic AMB resistance, and (c) associated with higher mortality rates [35]. A prospective international *Aspergillus terreus* study revealed that *A. terreus* s.s. was found to be the most frequently isolated species from clinical specimens (86.8%) followed by *A. citrinoterreus* (8.4%), *A. hortai* (2.6%), *A. alabamensis* (1.6%), *A. neoafricanus* (0.2%), and *A. floccosus* (0.2%). Frequencies of cryptic species vary with geographical origin [21]. A French study found similar occurrence of cryptic species [29], suggesting that cryptic species might be underdiagnosed in less experienced centers due to their morphological indifference.

*A. terreus s.s., A. citrinoterreus* and *A. hortai* are the most common found species in clinically samples [17,21,29]. These three species were found to exhibit highest growth and germination rates at 30°C and 37°C, which might partially explain their high frequency in clinical specimen [17,21,29]. *A. terreus s.s*. is the most common human pathogen within the section *Terrei*, even though, growth or virulence potential was similar to the cryptic species. Differences in frequencies in patients might mirror the higher abundance in the environment and subsequent higher exposure of patients at risk to conidia of *A. terreus* s.s. The ecological niches and epidemiology of cryptic *Terrei* remains to be understood in greater detail. However, such studies are still limited due to the necessity of molecular methods for species discrimination that are not ubiquitously available [17,21,29].

Possibly, *A. terreus s.s*. strains that have been re-cultured over many generations in the laboratory, underwent physiological changes during cryopreservation (degeneration and aging) and subcultivation on artificial media and these changes are disadvantageous in respect to host adaptation [32].

Adaptation to different micro-environmental stressors is necessary to allow germination and subsequent infection and strongly influences virulence potential. Here, we showed that all species are able to grow at conditions mimicking host conditions: low amount of nutrients, high temperatures, and reduced oxygen conditions. Interestingly, highest growth and germination rates were found at 37°C, which might be attributed to the saprophytic life style of these fungi and enables them to cause infections in immunocompromised hosts. Adaptation to 42°C (upper limit of growth temperature range) seems to be strongly strain dependent, indicated by great variations in colony diameters and high standard deviations within parallel experiments. In contrast, *A. fumigatus* seems to have a higher thermotolerance than *A. terreus*. Hagiwara et al. [36] found that *A. fumigatus* can cope with temperatures up to 60°C. No species-dependent difference was observed in hypoxic cultures, leading to the assumption that all species are similarly able to cause invasive disease, as also reflected by clinical data. Only *A. floccosus* has so far never been described to cause invasive disease [21,37].

A comprehensive study on the formation of AC showed that all 100 *A. terreus* isolates tested produced AC [22]. In this study, isolates were all assigned to the morphological species *A. terreus (A. terreus s.l.)*. We evaluated the formation of AC by the cryptic species and found that all species, except *A. floccosus* (*n* = 2), were able to produce AC in our experimental set-up. The ability to form AC within the host is one putative feature facilitating dissemination of *A. terreus* via the blood stream. In addition, the ability of phialidic *A. terreus* conidia to survive within host immune cells (macrophages and dendritic cells) might contribute to the dispersal to different body parts and subsequently disseminated infection. The persistence of *A. terreus* conidia is due to their resistance to (a) low pH and microcidial enzymes in lysosomes of macrophages, and (b) high pH and low nutrient availability inside dendritic cells. The intracellular persistence of viable *A. terreus* conidia in the lungs of healthy and immunocompromised mice without clinical symptoms enforces this hypothesis [26,27,33]. Here, neither phagocytosis of *A. terreus* conidia by, nor their fate within *Galleria* hemocytes was investigated and it certainly would be interesting to investigate if both types of *A. terreus* conidia could persist in hemocytes, and if differences between species of the section *Terrei* could be observed. Nevertheless, in case that the ability to persist within human immune cells is unique to *A. terreus* s.s., this might be one possible explanation for the higher abundancy of *A. terreus* s.s. in patient samples compared to the cryptic species.

Larvae of the greater wax moth, *G. mellonella*, have been widely used as alternative model to evaluate the virulence of fungal pathogens [38–40]. The larval size and anatomy enables precise injection of a defined number of conidia. Incubation at 37°C is possible and insects possess a cellular and humoral innate immune system with great similarities to the innate immune response of vertebrates [41]. Hemocytes were shown to exhibit strong structural and functional similarities with mammalian phagocytes, such as the recognition of pathogens via peptidoglycan recognition proteins, the induction of cell signaling through Toll, ImD, and Jak-STAT pathways and the expression of antimicrobial peptides [42,43]. Plasmatocytes and granulocytes, two types of *Galleria* hemocytes, were shown to phagocytose and kill microbes via NADPH dependent mechanisms, similar to mammalian neutrophils. Further, their function is repressed by secondary metabolites produced by *A. fumigatus*, gliotoxin and fumagillin, in a similar manner than the function of neutrophils [44,45]. A recent study highlighted significant parallels between the larval and mammalian response to aspergillosis, such as (a) an increase in immune cells after infection, (b) the ability to discriminate between the developmental fungal stages (resting spores, germinated spores, hyphae), (c) increased response to fungal pathogens when challenged with dead A. fumigatus conidia in an *ex vivo* assay, (d) similar capacity of tissue invasion and (e) increase in antimicrobial peptides [46]. Limitations of this alternative model are: the unavailability of an adaptive immune system, lacking understanding in the immune system and immune response of the larvae and the lack of standardized *Galleria* lines and experimental protocols [40]. However, for *A. fumigatus* a strong correlation between the larvae and murine models were observed in several studies [47,48]. One study even illustrating similar outcome in 4 different model system: larval model, pulmonary and systemic murine model and a keratitis model [49]. In one of our previous studies [31] we showed differences in virulence potential of AMB resistant and AMB susceptible isolates. Same results were obtained in murine studies [50], which highlights the transferability of *A. terreus* data in larvae to vertebrate models.

Overall, the larvae are a useful alternative *in vivo* model, especially to screen a large number of strains, and further helps incredibly to reduce the number of vertebrates in animal experiments.

Correlation between virulence potential and *in vitro* growth came to contrary conclusions. Petraitis *et al*. [51] demonstrated a positive correlation between virulence potential *in vivo* with species-dependent differences in germination rate *in vitro*. In a previous study, a positive correlation between higher virulence potential and faster germination and growth of AMB resistant *A. terreus* isolates was found when investigating a small strain set [31]. For *A. fumigatus* color mutants no positive correlation between faster germination and higher pathogenicity could be detected [52]. Overall, higher virulence potential correlated to better growth at 37°C in our study. Further, differences in virulence potential of individual strains were independent to the source (geographically and body site). A limitations of our study is that we only compared clinical isolates. Certainly, it would have been interesting to compare virulence of environmental isolates as well. From the data we have obtained in other studies [53,54], it is tempting to assume we would not have detected correlation between virulence potential and the origin of the strains (clinical or environmental). Nevertheless, one study carried out in *Galleria mellonella* showed that clinical *A. fumigatus* isolates were found to be more pathogenic than environmental isolates [55].

Our results obtained from comparing virulence potential of AC and PC highlights the importance of screening large strain sets to get a broad picture on virulence potential. Deak et al. [22,23] showed that AC of one single isolate are superior in germination, metabolism and cause higher inflammatory response in lungs of infected mice compared to PC. Regarding virulence data obtained in our study, we conclude that these differences might be strain specific and need to be evaluated in more detail. Further evaluations are going on deciphering species depending differences in cell surface of AC, morphology or ergosterol content.

The production of secreted secondary metabolites, enzymes, or toxins might also play an important role in the ability to cause infection [56]. *A. terreus* species were shown to produce a wide variety of secondary metabolites, which differ in-between species [9]. According to our findings, species-dependent production of different metabolites, or combinations thereof, seems not to play a major role in the *Galleria* model, where all species were able to cause death to the larvae. To understand their role in pathogenicity, the production of secondary metabolites, such as gliotoxin or citrinin, has to be studied in detail. Unless this is done, we can only speculate about their contribution to virulence potential.

Taken together, we postulate that, beyond host related factors, virulence potential is a strain- rather than species-specific feature. Data from the current study and previous studies on AMB resistance [17] and azole-resistance rates [20,21] suggest that from a clinical point of view a discrimination of the cryptic species is not necessary in a routine laboratory, since all cryptic species behave similar in terms of: (a) virulence potential, (b) stress tolerance (oxygen, nutrients, temperature), (c) AMB resistance, (d) azole resistance rates and (e) account only for a minor fraction of aspergillosis cases. From a clinical point of view, pathogenicity of *A. terreus s.s*. is indifferent from cryptic species and could be referred to as *A. terreus* s.l. in clinical routine diagnostic laboratories.

## Materials and methods

### Fungal strains and growth conditions

A total of 73 *Terrei* isolates, derived from clinical specimen, were used (details on isolates can be found in Table S1). Isolates were collected previously in the frame of a ISHAM-ECMM-EFISG TerrNet Study group (www.isham.org/working-groups/aspergillus-terreus) and were isolated in different clinics in several countries [17,21].

Species identification was already done in the TerrNet Study [17] and was performed by sequencing the internal transcribed spacer region (ITS 3–4), gene regions of β-tubulin, calmodulin, enolase and cytochrome C. No further molecular analysis of strains was carried out in this study. Fungi were grown on Sabouraud-Agar for 7 days at 37°C to obtain phialidic conidia. Phialidic conidia were harvested in spore suspension buffer (0.9% NaCl, 0.01% Tween 20) or insect physiological saline (IPS; 150 mM NaCl, 5 mM KCl, 10 mM EDTA and 30 mM sodium citrate in 0.1 M Tris–HCl, pH 6.9), washed and filtered through a 40 µm cell-strainer (BD, Heidelberg, Germany). Number of spores was determined by hemocytometer.

Accessory conidia were obtained as described before [57] with the following changes: 10^8^ freshly harvested phialidic conidia were added to 250 ml Sabouraud media and incubated at 28°C, 220 rpm for 5 days. Formation of AC was determined microscopically before culture was transferred into a sterile bottle and AC were harvested by vigorous shaking and ultrasound. AC were separated from mycelia by filtration (40 µm cell strainer) and repeated washing by centrifugation.

### Radial growth assays

Radial growth was determined on different media (*Aspergillus* complete medium (ACM) [31] and RPMI_1640_, in different temperatures and in different oxygen conditions. Therefore, 10^4^ spores of the individual strains were dotted on the respective agar plates and incubated at the determined conditions. After 48 h colony diameters were measured and growth documented visually. Experiments were carried out in three parallels and repeated twice. For growth assays in hypoxic conditions cultures were grown at 1% O_2_ (Biospherix C-Chamber & Pro-Ox controller, USA).

### Evaluation of spore germination

To determine germination rates, 100 µl of conidial suspension (1*10^5^/ml) were placed in a 96 well microdilution plate and incubated for 8 h. Germination rate for each isolate was determined microscopically by calculating the percentage of conidia with germ-tube formation out of 100 randomly selected conidia. Assays were repeated three times with two parallels each time.

### *Virulence studies in* G. mellonella

Sixth instar larvae of *G. mellonella* (SAGIP, Italy) were stored in wood shavings in the dark at 18°C prior to use. Larvae weighing 0.3–0.4 g were selected for experimental use. Inoculum preparation, larval infection and monitoring of survival were carried out as described previously [31]. Larvae injected with sterile insect physiological saline (IPS; 150 mM NaCl, 5 mM KCl, 10 mM EDTA and 30 mM sodium citrate in 0.1 M Tris–HCl, pH 6.9) [58] and untouched larvae served as controls. Larvae were infected 1*10^7^ or 10^6^ conidia per larva of each *A. terreus* isolate tested. Survival was monitored in parallel at 30°C or 37°C over 144 h. In each experiment 20 larvae per sample were injected, the experiments repeated three times and the average survival of all 60 larvae was calculated. To determine whether accessory conidia differ in their virulence potential compared to phialidic conidia larvae were infected with 10^6^ conidia of each type, respectively and incubated at 37°C.

### Histology of larvae

Infected larvae plus control larvae (injected with IPS), incubated at 37°C, were injected with formalin and conserved as a whole in formalin for 10 days; then embedded in paraffin, longitudinally cut at 5 µm and stained with Grocott´s silver stain to detect fungal elements [54,59].

### Statistical analysis

All experiments were performed on at least three independent occasions. Results were expressed as the mean ± SD. Survival rates were evaluated by using Kaplan Meier survival curves and analyzed with the log rank (Mantel-Cox) method utilizing GraphPad Prism software. Further, Tukey´s, Sidak´s multiple comparison (ANOVA) and t-test were used to determine differences. Correlation was evaluated according to Pearson. Differences were considered significant at *p ≤ 0.05.

## Transparency declaration

C. L-F has received grant support from the Austrian Science Fund (FWF), MFF Tirol, Astellas Pharma, Gilead Sciences, Pfizer, Schering Plough, and Merck Sharp & Dohme. She has been an advisor/consultant to Gilead Sciences, Merck Sharp & Dohme, Pfizer, and Schering Plough. She has received travel/accommodation expenses from Gilead Sciences, Merck Sharp & Dohme, Pfizer, Astellas, and Schering Plough and has been paid for talks on behalf of Gilead Sciences, Merck Sharp & Dohme, Pfizer, Astellas, and Schering Plough. All other authors have no conflicts of interest to declare.
