# Supplementary figures and images for: Cryptic species of Aspergillus section Terrei display essential physiological features to cause infection and are similar in their virulence potential in Galleria mellonella
Source: Virulence. 2019 Jun 6;10(1):542–54. doi: 10.1080/21505594.2019.1614382 (PMC6592363; doi:10.1080/21505594.2019.1614382)

Figure S1

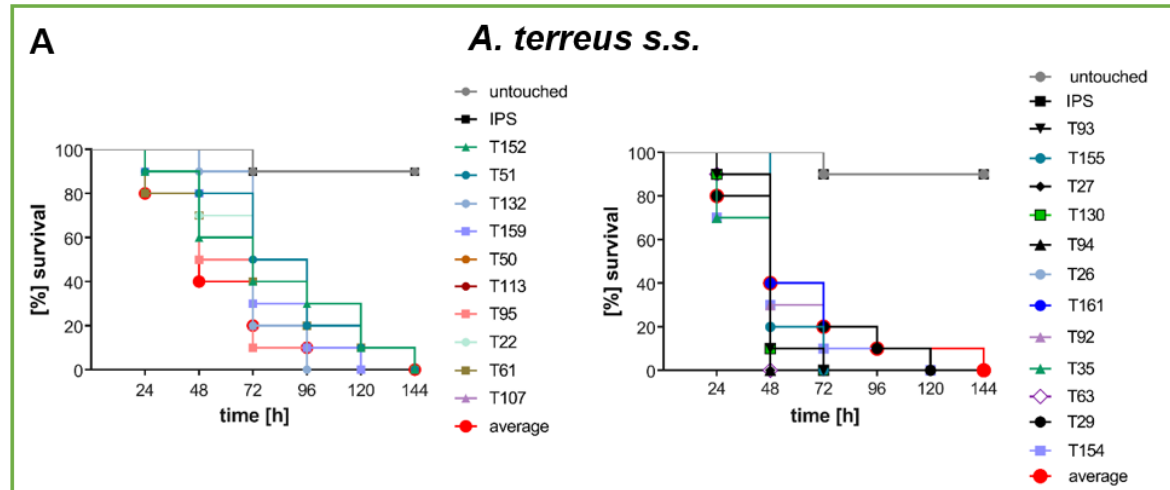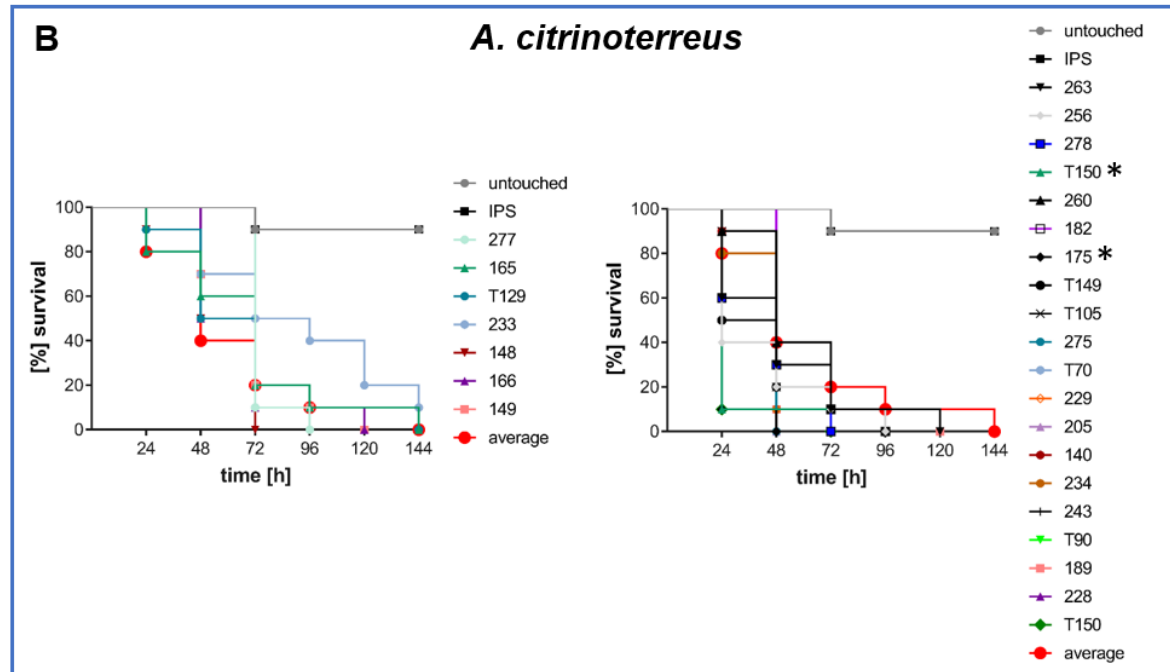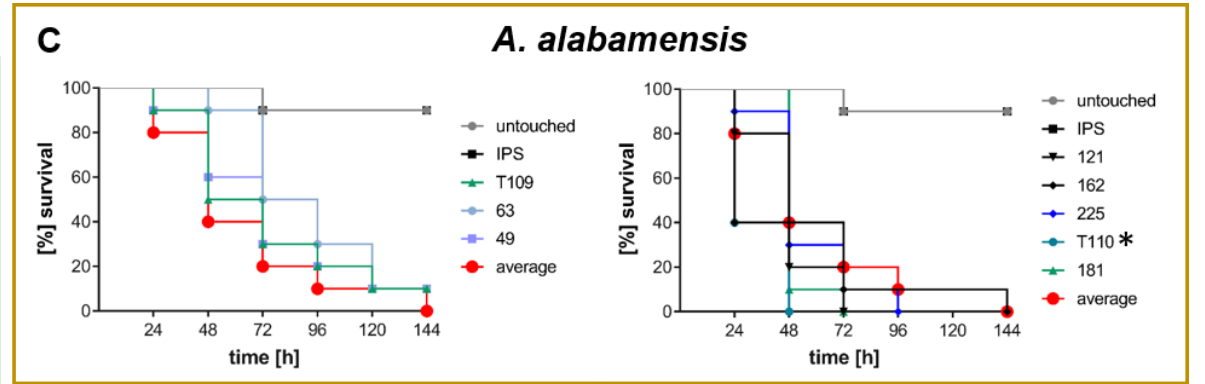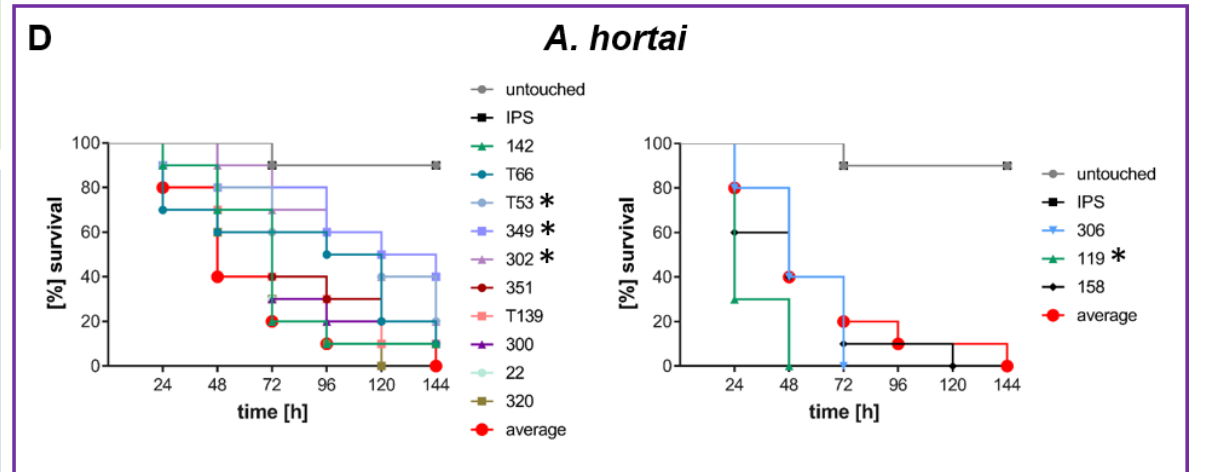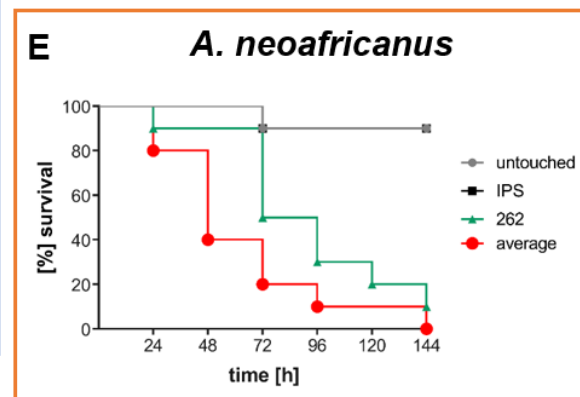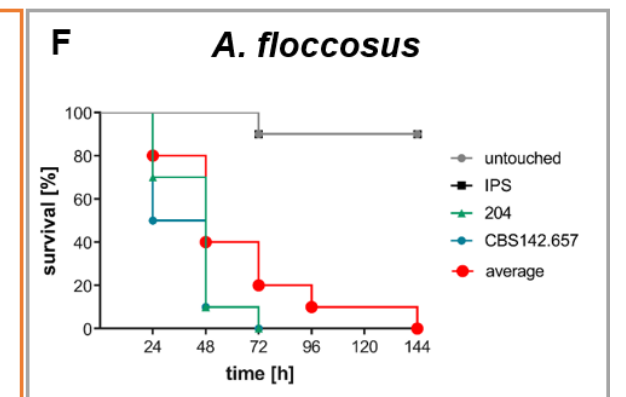

**Figure S2**

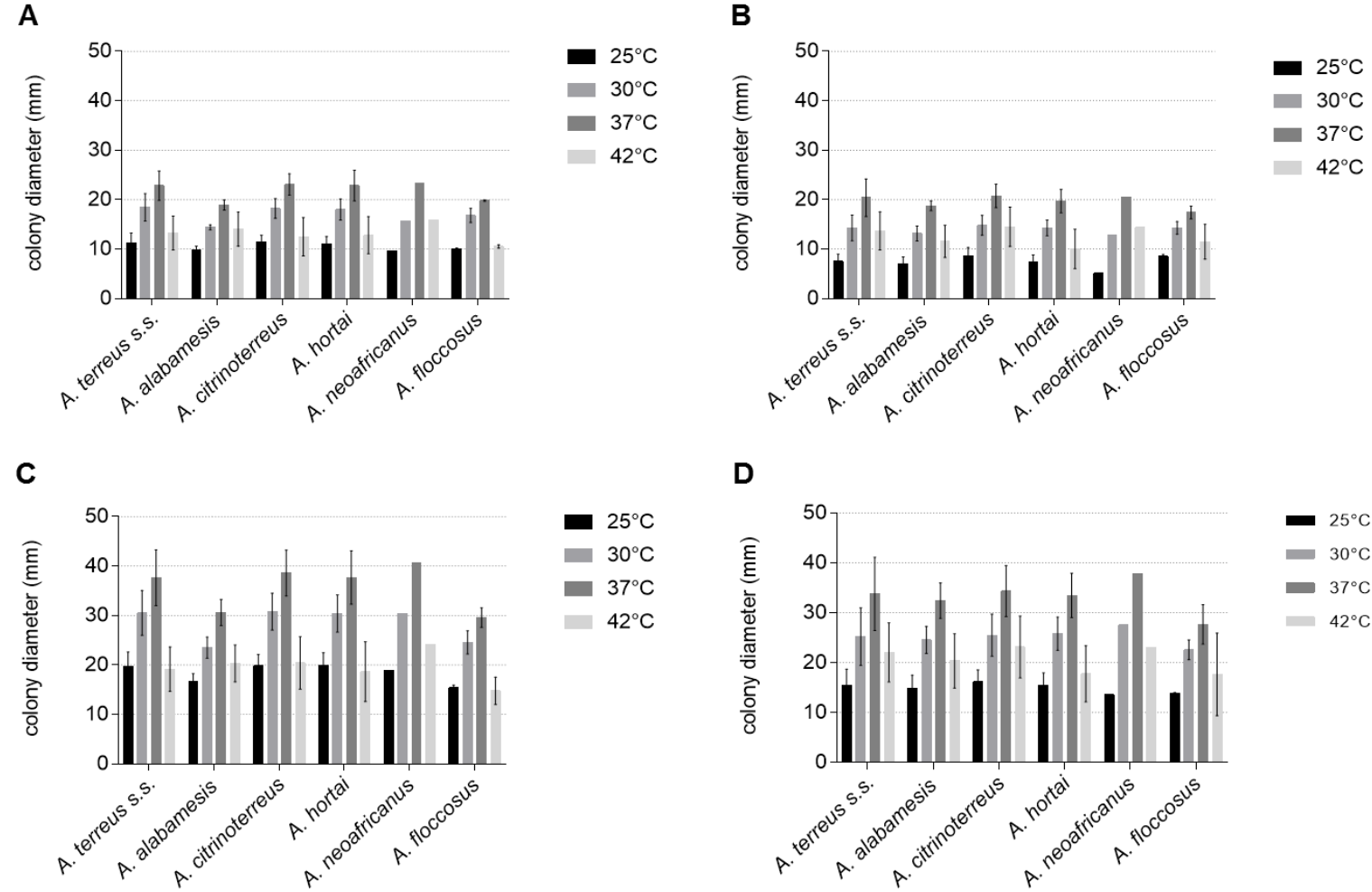

### Figure S3

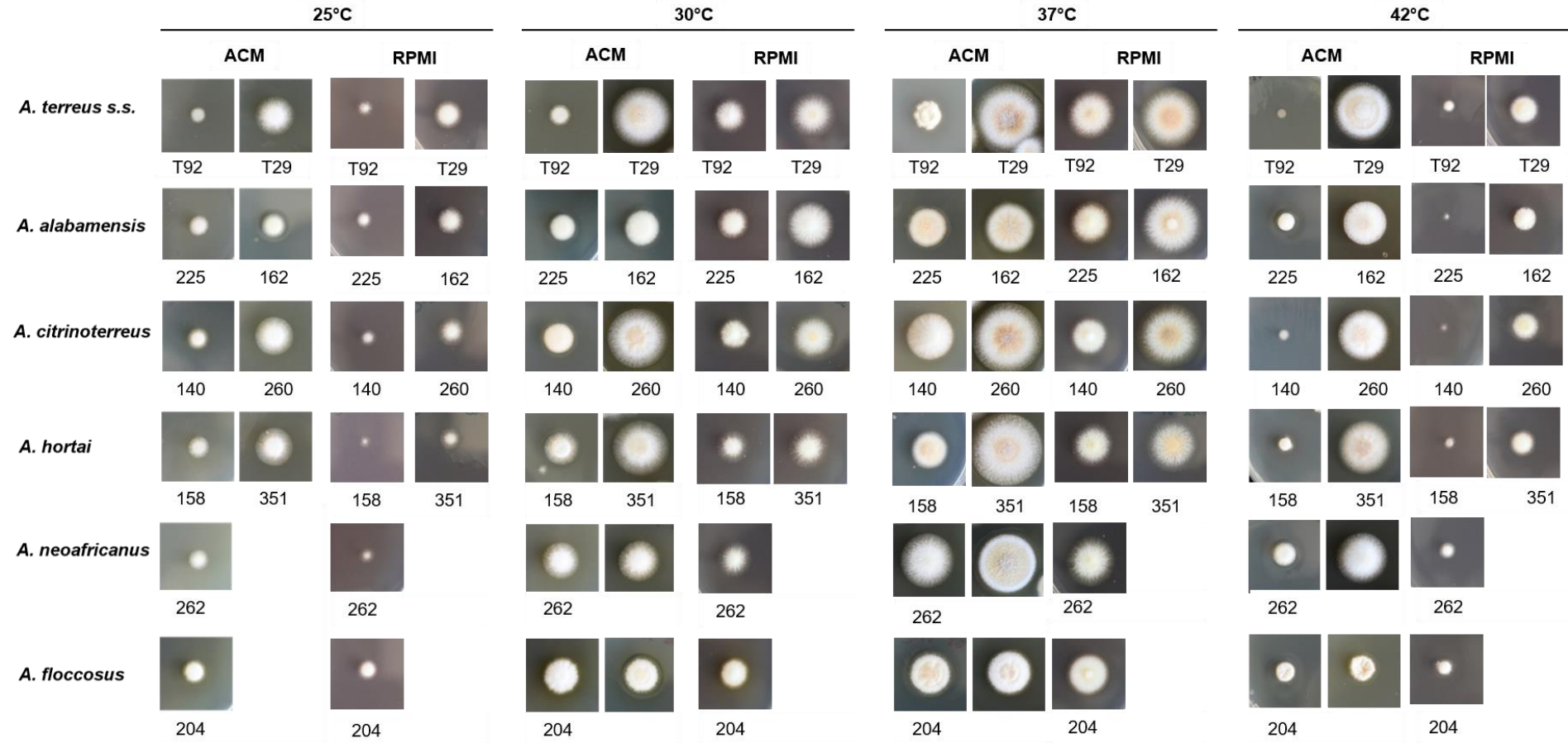

Supplement: Supplemental Material [file kvir-10-01-1614382-s001.pdf]
